# Complex relationships between soybean trade destination and tropical deforestation

**DOI:** 10.1038/s41598-023-38405-1

**Published:** 2023-07-12

**Authors:** Ramon Felipe Bicudo da Silva, Emilio F. Moran, James D. A. Millington, Andrés Viña, Jianguo Liu

**Affiliations:** 1 Department of Fisheries and Wildlife, Center for Systems Integration and Sustainability, Michigan State University, East Lansing, MI 48823 USA; 2 Center for Environmental Studies and Research, State University of Campinas, Campinas, 13083-867 Brazil; 3 Center for Global Change and Earth Observations, Michigan State University, East Lansing, MI 48823 USA; 4 Department of Geography, King’s College London, London, WC2B 4BG UK; 5 Department of Geography and Environment, University of North Carolina, Chapel Hill, NC USA

**Keywords:** Environmental impact, Sustainability

## Abstract

Over the last few years, understanding of the effects of increasingly interconnected global flows of agricultural commodities on coupled human and natural systems has significantly improved. However, many important factors in environmental change that are influenced by these commodity flows are still not well understood. Here, we present an empirical spatial modelling approach to assess how changes in forest cover are influenced by trade destination. Using data for soybean-producing municipalities in the state of Mato Grosso, Brazil, between 2004 and 2017, we evaluated the relationships between forest cover change and the annual soybean trade destination. Results show that although most of the soybean produced in Mato Grosso during the study period (60%) was destined for international markets, municipalities with greater and more consistent soybean production not destined for international markets during the study period were more strongly associated with deforestation. In these municipalities, soybean production was also significantly correlated with cattle and pasture expansion. These results have important implications for the sustainable management of natural resources in the face of an increasingly interconnected world, while also helping to identify the most suitable locations for implementing policies to reduce deforestation risks.

## Introduction

There is increasing recognition of the value of studying coupled human and natural systems as intricate entities embedded within broader systems (e.g., metacoupled human and natural systems^1–3^) and that demand for natural resources shapes land use/cover change trajectories worldwide^4–6^. Among the list of land use/cover changes with global impacts, deforestation and its resultant conversion of natural forests to pastureland, cropland and built-up areas is recognized as a key sustainability challenge^7,8^. In tropical regions, the drivers of deforestation vary according to different political, social, economic and environmental contexts. In the case of Brazil, the production of agricultural commodities (including soybean and beef) as well as land speculation, constitute a major driver^9^. Although beef production (19% of which is exported, making Brazil the World’s largest exporter^10^) is considered the most important driver of deforestation, soybean production has also become a major driver in recent decades^11^. Given that around 80% of the soybeans produced in Brazil is exported, mainly to China and Europe^12^, it has been the focus of attention of international research and policy interventions^6,8–10^.

The globally interconnected supply chains have fostered the need to develop global environmental governance systems aimed towards sustainable production chains^13–16^. Widely-recognized environmental degradation by the tropical timber trade to supply major importers (e.g., European Union, US, Japan) has prompted the emergence of eco-certification systems to promote sustainability in this supply chain^15,17^. Multi-stakeholder sectoral standards such as those of the Forest Stewardship Council (FSC) and the Soy Moratorium (SM) are complementary to local/national policies in promoting positive environmental outcomes^8,13,15^. The SM (implemented in 2006) is a supply chain initiative to mitigate deforestation in the Brazilian Amazon by preventing international companies from purchasing soybean produced on land deforested after 2008^6^. A number of studies have evaluated the impacts of global agricultural commodity supply chains (e.g., beef, palm oil, and soybean) on tropical deforestation and related environmental impacts^6,18–20^. For instance, Pendrill et al.^21^ have shown that the international agricultural commodity trade is responsible for up to 39% of greenhouse gas emissions related to tropical deforestation. Malaysia and Indonesia, responsible for 90% of the global exports of palm oil^22^, lost around 45 Mha of natural forest cover, 33% (15 Mha) due to the expansion of oil palm plantations^23^. Yet few studies have evaluated the influence of supply chain interventions on the spatio-temporal dynamics of natural forests^22^, nor whether agricultural production for international markets plays a different role from that of production for domestic markets in driving landscape change. Additionally, for Brazil, zu Ermgassen et al.^10^ showed that agricultural commodities destined for international markets are more likely to be sourced from municipalities with a more consolidated land use system with few forest remnants, suggesting that municipalities with more extensive forest cover (more likely experiencing greater deforestation) may be producing agricultural commodities destined more for national than for international markets. However, there has been no quantitative analysis addressing the role of soybean production for international vs. national markets on deforestation. This study addresses this knowledge gap, while also advances previous assessments of the impacts of agricultural supply chains on tropical forests^10,24,25^.

A previous study on soybean trade ‘stickiness’ (a measure of stability between soybean suppliers and market destinations through time) between logistic hubs in Brazil and international markets^25^ showed that more temporally stable supply chain configurations, which are also usually more committed to policies of zero deforestation (e.g., Soy Moratorium), are less associated with deforestation risks. This suggests that besides the continuous increase in soybean production, inter-annual changes in market destination are also related with the negative environmental outcomes of the soybean trade (e.g., deforestation). Given the lack of knowledge and attention from stakeholders in Brazil regarding the potential effects of trade destination, here we introduce an empirical evaluation of the association between soybean trade destination and deforestation. To this end, this study analyzed deforestation at the municipality level between 2004 and 2017 throughout the state of Mato Grosso, Brazil. By evaluating the environmental outcomes of the trade destination of an important global agricultural commodity, this study assesses a frequently neglected factor affecting environmental change^25,26^ within the context of increasingly interconnected global supply chain governance systems^27^. To this end, our study addresses three important and interrelated questions: (*i*) How does deforestation in relation to soybean production is influenced by trade destination? (*ii*) Are municipalities with a majority of their soybean production destined for international markets more or less associated with deforestation? (*iii*) Considering possible inter-annual fluctuations in the total proportion of soybean destined for international markets, is there a significant association between the degree of market instability and deforestation? Finally, the study also explores the association between the production of soybean and beef (the latter associated with pasture expansion), and farm size, which are important features of agricultural frontiers in Brazil linked to deforestation^10,28^.

## Results

### Land use change

**Figure 1:**
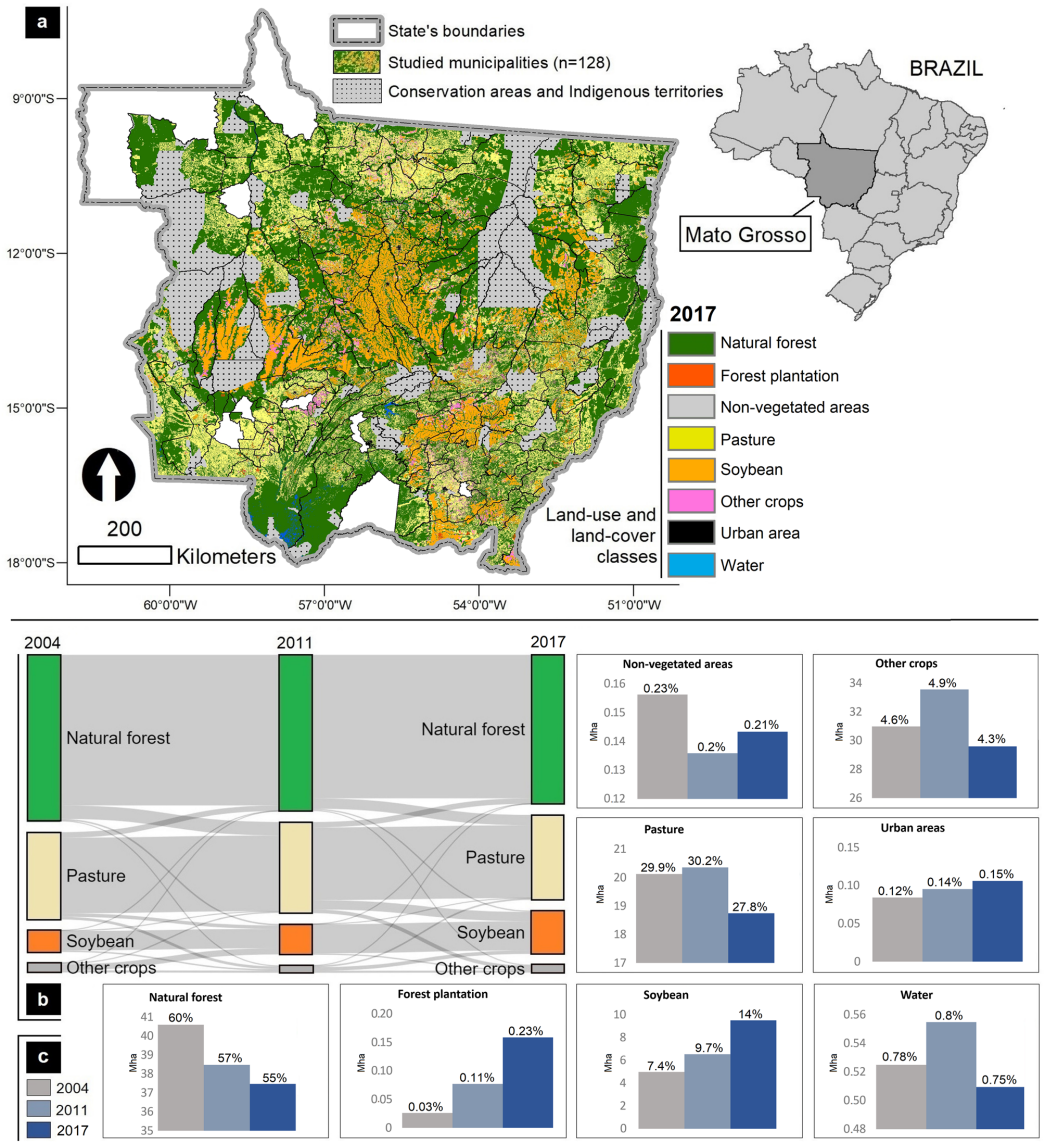
Land-use/cover (LULC) data of Mato Grosso State. (**a**) Map of the study area (state of Mato Grosso excluding conservation areas and indigenous territories) representing the spatial distribution of land use/cover classes in 2017 (these data were derived from MapBiomas *v7.0*, https://mapbiomas.org/). (**b**) Sankey diagram highlighting land transitions in major land use/cover classes observed in the state of Mato Grosso, Brazil, between 2004 and 2017. (**c)** Bar charts present all land use/cover classes and their respective areas (and percentage in relation to the total study area) for the years 2004, 2011, and 2017.

Between 2004 and 2017 the areal extent of ‘natural forest’ in the study area (Fig. 1a) was reduced by 8% (from 40.6 Mha in 2004 to 37.4 Mha in 2017), while the area under soybean increased by 90% (from 4.9 Mha to 9.5 Mha). The ‘other crops’ class exhibited more stability, changing from 3.1 Mha in 2004 to 2.9 Mha in 2017, or a loss of 4.5%, while the class ‘pasture’ exhibited a slight decrease of 6%. For instance, between 2004 and 2011, ‘other crops’ was the LULC class with the greatest replacement by ‘soybean’, while during the period from 2011 to 2017, ‘pasture’ exhibited the greatest percent of transition to ‘soybean’ (Supplementary Material, Tables 1 and 2). The landscape metric ‘percentage of the landscape’ shows that the ‘natural forest’ was the largest land use/cover class, followed by ‘pasture’ and ‘soybean’, respectively (Fig. 1c).

The 2011 data in the Sankey diagram (Fig. 1b) highlights that throughout the entire period studied there were no changes in the direction of major LULC transitions, although the intensities of these transitions did change. The main LULC classes (i.e., covering largest areas) had similar transition rates from ‘natural forest’ to ‘pasture’ in both periods (2004–2011, and 2011–2017), while ‘soybean’ mostly replaced ‘pasture’. These results support the idea that while ‘pasture’ expansion replaces ‘natural forest’ (i.e., directly driving deforestation), by replacing pasturelands, ‘soybean’ expansion indirectly pushes deforestation elsewhere through the formation of new ‘pasture’ areas over forested lands^29^.

### Soybean trade destination

**Figure 2:**
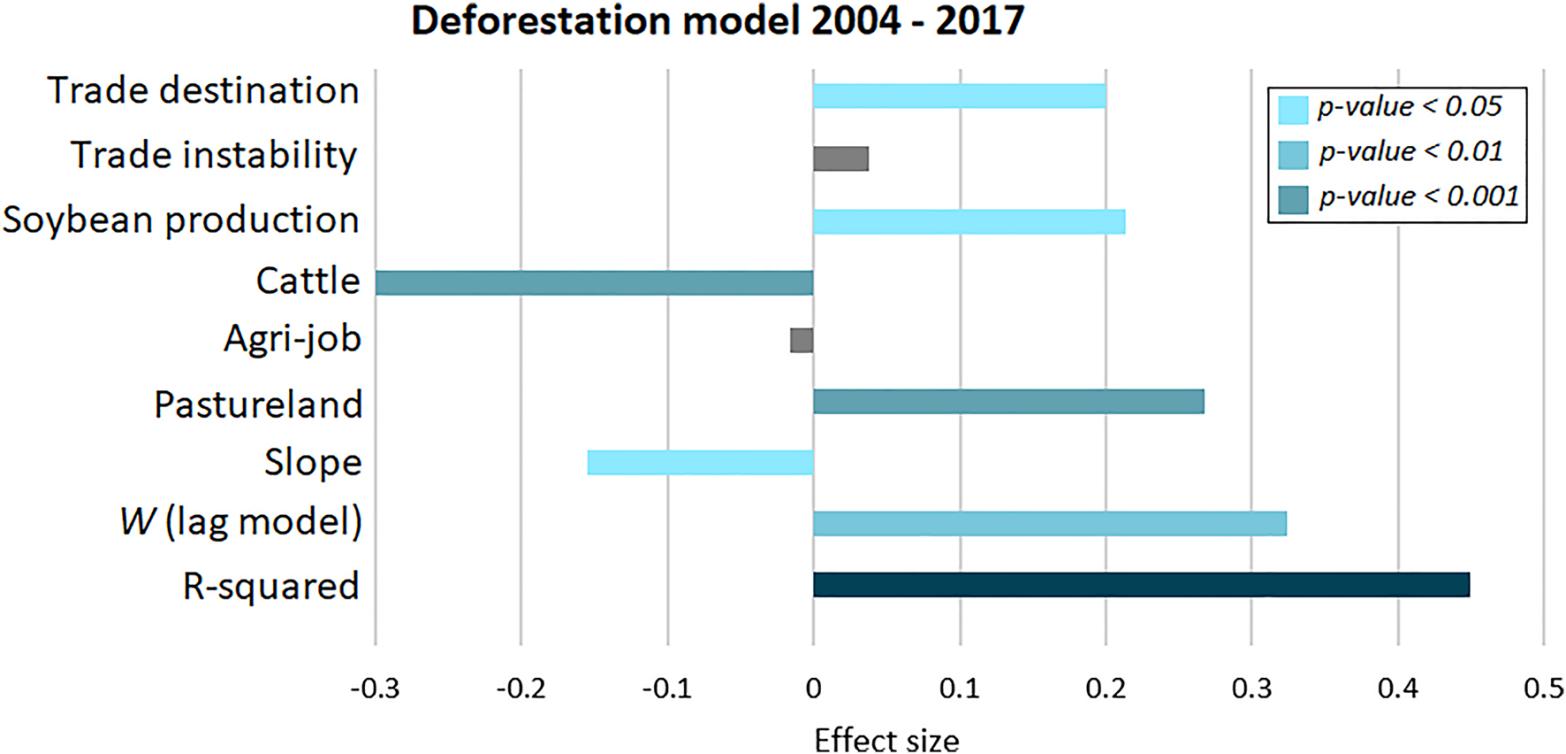
Spatial regression model relating the proportion of soybean production destined for international markets and deforestation across soybean producing municipalities of Mato Grosso, Brazil (n = 128). *W* is the spatial lag term of the dependent variable (its coefficient parameter, rho, reflects the spatial dependence inherent in the data). Regression results are presented in Supplementary Material, Table 3—Spatial regression model results. The trade destination variable in this model represents the new variable created by this study based on the TRASE soybean exports and IBGE production datasets.

We developed a spatial regression model for the period of 2004–2017 to assess the relationships between soybean production and deforestation. The model assessed the association between the proportion of the total soybean production not destined for international markets (from 0 to 100%, where 0% means soybean production entirely destined for international markets over the time period evaluated) with deforestation (Fig. 2). Lagrange Multiplier (LM) tests found significant p-values for spatial autoregressive models (SARlag). We did not find explanatory variables with VIF values greater than 5, indicating an absence of multicollinearity issues.

**Figure 3:**
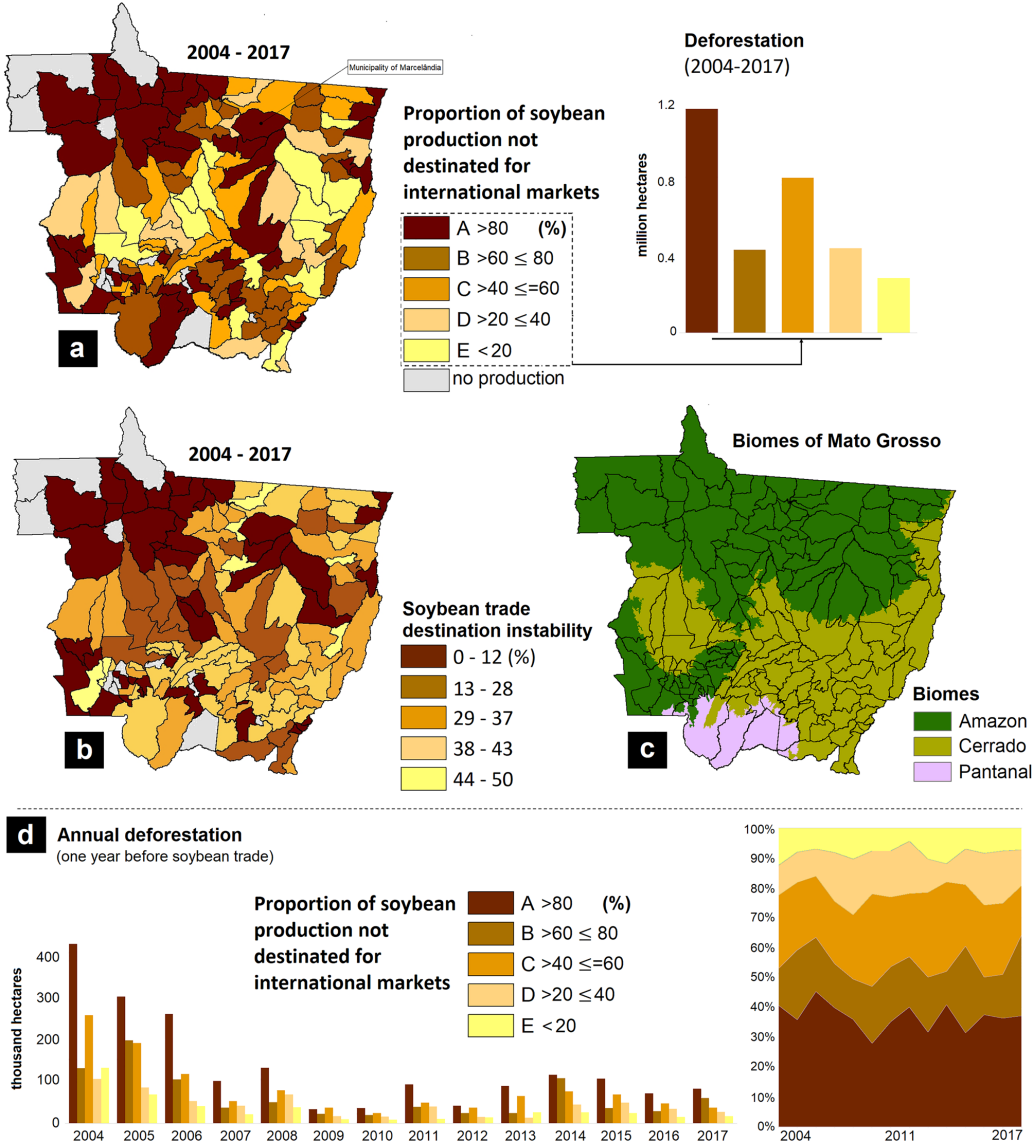
Soybean trade destination dynamics. (**a**) Choropleth map of the municipalities in Mato Grosso, Brazil representing the proportion of soybean production not destined for international markets along the 2004–2017 period. The complement reflects production for international markets. (**b**) Choropleth map of the municipalities in Mato Grosso, Brazil representing soybean ‘trade destination instability’ along the 2004–2017 period. Trade destination instability is defined by the standard deviation in market destination, where lower values represent less instability (i.e., less variation along the time period). (**c**) Spatial distribution of the Brazilian biomes within the state of Mato Grosso, Brazil. (**d**) Total deforestation over the previous year—data grouped according to the same classes determined by the proportion of soybean production not destined for international markets.

Our regression result shows that municipalities with a higher proportion of soybean production not destined for international markets exhibit statistically significant associations with deforestation (“Trade destination”—Fig. 2), a situation observed annually during the entire study period (Fig. 3d). These results address our first and second questions posited in the Introduction. We also found a strong and significant negative correlation between the proportion of soybean production not destined for international markets and ‘trade destination instability’ (rho -0.49, p < 0.001). This result reveals that municipalities with a lower proportion of soybean production destined for international markets tend to exhibit lower trade instability (i.e., lower SD values; Fig. 3a,b). To provide the spatial (geographical) context of the distribution of trade destination of soybean production, we generated choropleth maps of the proportion of soybean production not destined for international markets (Fig. 3a) and trade ‘instability’ (Fig. 3b) using Natural Breaks. Figure 3d demonstrates that municipalities with higher annual proportion of soybean production not destined for international markets (> 80%), exhibiting a greater association with deforestation over the previous year. The group of municipalities with a higher proportion of soybean production not destined for international markets (> 80%; class A) contributed 37% of the deforestation that occurred between 2004 and 2017 (Fig. 3a), while those with a high proportion destined to international markets (class E) contributed only 9%. Additionally, municipalities of class A had an average SD at 7% (i.e., mean value of the SD—the lower the SD, the lower the trade instability), while classes B, C, D, and E had values of 35%, 38%, 35%, and 20%, respectively. These key results show that, despite the variability observed over time in market destination and ‘trade destination instability’, municipalities producing more soybean not destined for international markets were responsible for the largest share of deforestation in the state of Mato Grosso, Brazil. It is important to note that our dataset includes 128 municipalities in the state of Mato Grosso that produce soybean, while Trase has data on trade for 112 municipalities in Mato Grosso. Hence, by using both sources of data we were able to develop a dataset that accounts for municipalities producing soybean not destined for international markets.

Given the major land change transitions involved in natural forest, pasture and soybean areas (Fig. 1b), we analyzed the correlation between cattle statistics (number of animals and cattle density) and pasture area vs. proportion of total market not destined for international markets and soybean trade destination instability. Results indicate that the proportion of soybean production not destined for international markets was significantly correlated with cattle herd changes during the 2004–2017 period (0.25, p < 0.05), with mean cattle density (rho 0.25, p < 0.01), and with the expansion of pasture area (rho 0.26, p < 0.05). These results suggest that municipalities with a larger proportion of soybean production not destined for international markets (during the 2004–2017 period) also exhibit a higher expansion of pasture and cattle production (Supplementary Material*—*Fig. 1). This reinforces the spatial association observed in frontier areas (with more intense deforestation) dominated by indirect changes in land use in response to soybean expansion (i.e., land use change from natural forest to pastureland, followed by changes from pastureland to soybean production). In addition, a significant negative correlation was found between the average soybean farm size per municipality with the proportion of soybean production not destined for international markets (rho -0.40, p < 0.001). This suggests that the soybean produced in municipalities with smaller soybean farms tend to be less destined for international markets than the one produced in municipalities with larger soybean farms (Supplementary Material*—*Fig. 2). Similar results were obtained for soybean ‘trade destination instability’ (rho 0.21, p < 0.05). All correlation test results are presented in Supplementary Material, Table 4—Correlations.

## Discussion and conclusion

Global awareness of the relationship between international commodity flows and land use/cover dynamics have fostered the emergence of governance systems to achieve sustainable production^12^, with a special focus on telecoupled systems^14,15^. For instance, in 2018 around 40% of the Brazilian soybean exports were under a governance commitment of zero deforestation^30^. It has also been reported that only six large traders were responsible for nearly 58% of the Brazilian soybean exports, while the emergent soybean market for small and medium traders exhibits more deforestation risks, as they are more likely operating in agricultural frontiers^30^. Furthermore, small/medium players, which are usually non-signatories of sustainability commitments^31^, tend to produce soybean that is not destined for international markets (e.g., for animal feed, recently boosted by increasing Brazilian meat production^30^). According to the *Mato Grosso Institute of Agricultural Economics*^32^, around 40% of soybean production is not destined for international markets (the same shown in our dataset derived from Trase and IBGE sources for the 2004–2017 period). From this amount, ca. 75% is processed in-state while ca. 25% is sent to other Brazilian states^32^. Zu Ermgassen et al.^10^, studying beef exports, also argued that in Brazilian municipalities with a lower participation in international markets or expanding agriculture, there is a higher likelihood of negative environmental outcomes, something we empirically demonstrate here using the soybean trade as the main focus. But here we call attention to a potential different effect, namely the marginalization of local markets and small/medium enterprises in favor of global trading companies and larger farming operations through supply chain interventions (which may be conveniently organized by the latter).

In Brazilian agricultural frontiers, the likelihood of deforestation has been found to be greater in smaller farms (over 2011–2016^26^). The lower likelihood associated with large farms can be attributed to easier monitoring and sanction (e.g., against international sustainability supply chain agreements) given their integration with international markets—and thus more susceptible to international pressure to avoid deforestation. Our study brings new information to this debate by showing that municipalities with small/medium average soybean farm sizes^33^ tend to be significantly associated with a higher proportion of soybean production that is not destined for international markets and with a lower trade destination instability. This is because such farms are less constrained by supply chain agreements^10,30^. Our map of soybean farm sizes (Supplementary Material—Fig. 2) indicates that small/medium farms are mainly located to the northwest and southwest areas of Mato Grosso (i.e., in the Amazon and Pantanal biomes—Fig. 3c), thus, more distant from the largest centers of soybean production (i.e., the Cerrado biome—Fig. 3c). Soybean production in the latter is more destined for international markets (Fig. 3a). Additionally, the costs associated with the integration into sustainability agreements or the adoption of sustainable practices can be higher for small than for large producers. Thus, the largest producers have a higher probability of generating more positive environmental outcomes, as they can access more exporting channels but are also exposed to higher pressures (while also receive more economic incentives) to adopt environmentally friendly practices (including lowering deforestation risks^34,35^).

By taking a multi-level perspective in our modelling approach, it was possible to evaluate the impacts of market destination on deforestation risk. Previous studies addressing deforestation risk have assessed the impacts of the amount of agricultural commodities being produced in a given locality^10^, or assessed the effects of a telecoupling process where changes in land use/cover are attributed to the flows of agricultural commodities for international markets^24,36,37^. Our study evaluated the association between trade destination and deforestation. Results from our model reveal that municipalities with a higher proportion of soybean production not destined for international markets (which tend to have lower ‘trade destination instability’) experienced more intense changes in natural forest cover over the time period evaluated (2004–2017). This is remarkable given that while soybean production for international markets increased 198% during the study period, production for other markets only increased 42%.

Previous studies have shown that sustainability agreements significantly influence supply chains in telecoupled systems^15,24^. Our results suggest that environmental governance initiatives that shape soybean production for international markets may be contributing to more sustainable international telecoupled systems, as demonstrated by zu Ermgassen et al.^24^. These authors found decreasing rates of deforestation in the Brazilian Amazon biome related to zero deforestation commitments. However, negative spillover impacts in the form of soybean production not destined for international markets, which in our study area tend to occur in frontier regions (Fig. 3a), are more likely to undergo deforestation and landscape change. As shown in Fig. 3d, municipalities with the highest percentages of their total production destined for international markets exhibited deforestation at higher rates during the years before 2008 (the year set by the SM), probably to allow production increases during the following years with no further deforestation. This has largely resulted in the decoupling between deforestation and soybean expansion in recent periods^24,38^. However, at the same time, deforestation remained high in municipalities with lower soybean production and whose production was not destined for international markets (Fig. 3d). Even in the presence of public policies aimed at environmental control (e.g., Brazilian Forest Code), in countries like Brazil where surveillance and law enforcement tend to be inefficient, many offenders tend to continue with their environmentally negative practices^39,40^. In contrast, where public policies are reinforced by the complementary role of (international) supply chain agreements (e.g., eco-certifications^8,13,15^), environmental outcomes tend to be more encouraging. This is supported by our results (Fig. 2), which indicated that municipalities with more/less production destined to international markets exhibited different environmental outcomes. For instance, while the SM decelerated deforestation in the Amazon, it boosted deforestation in the Cerrado biome (through spillover effects^6^). Nevertheless, our study has found progress in reducing deforestation rates, albeit significantly correlated with market destination. Here we advance previous knowledge by demonstrating that soybean production regions that are not committed to the production for international markets exhibit significantly higher deforestation pressures, including in the Amazon biome—where some deceleration in deforestation rates has been reported, albeit mostly in areas with production destined for international markets, and thus more committed to sustainability agreements^24^.

In addition, indirect land use changes due to soybean production following the replacement of natural forests with pasturelands is also an important factor^41^. Results from our correlation analyses highlight a significant association between the proportion of soybean production not destined for internal markets and cattle herd and pastureland expansion, reinforcing the land change processes noted in Fig. 1b (i.e., indirect land use change). These results also highlight that in the more active agricultural frontiers of Mato Grosso, soybean expansion has developed mainly to supply local/regional (within country) markets. Hence, while beef production is considered a major factor of deforestation in Brazilian landscapes^24,28^, we argue that producers have taken this pasture expansion as a “window of opportunity” to expand soybean production for less environmentally stringent market standards. As noted by zu Ermgassen et al.^24^, if non-compliant soybean farmers still clear land for production and decide to sell soybeans to non-committed traders, supply chain agreements will fail. Thus, while the international market has dominated the purchase of soybean produced with lower deforestation risks in more consolidated production zones^24^, this has left small trading companies and new and small producers—i.e., less integrated with international supply chains—to supply local/regional (within country) markets. In addition, those companies may directly purchase soybean from recently deforested areas in the Amazon to sell it to large trading companies, in a way to avoid surveillance and sanctions set by sustainability agreements, what has been known as soybean “washing”—something already observed in Marcelândia^42^ (municipality group A, Fig. 3a). In this case, and given the lack of trade information regarding the Brazilian internal market and difficulties to fully implement monitoring actions for the SM^42^, we highlight that within-country trade can potentially (not exclusively) be used as a step to “clean” soybean from deforestation before delivering it to international markets.

While the international market of agricultural commodities has received much attention from researchers and stakeholders to address deforestation and to foster more sustainable agricultural practices^10,24,25^, the influence of domestic markets tends to be neglected. Our findings using soybean as the main agricultural commodity may also be mirrored by other agricultural and forestry products, in which different market destinations exert differential environmental outcomes^43^. For instance, it has been found that the commercial production of eucalyptus plantations in Brazil, tied to international cellulose pulp markets, are fostering natural forest recovery^15^, while the opposite (i.e., natural forest loss) is occurring in areas with eucalyptus production that is more oriented towards domestic markets^44^. This suggests that more attention should be given to this effect, not only in supply chain agreements but also in environmental governance systems, particularly if there is an increase in agricultural production not only in Mato Grosso but elsewhere in Brazil and the world.

Although numerous efforts have proved effective to some degree at mitigating deforestation and environmental degradation within international agricultural supply chains^24,25,38^, national supply chains still lack focused policy-governance approaches. In addition, while historical attention has been given to the international soybean market^45–47^ and impacts of international trade^48^, there is still a lack of knowledge about how companies and producers operate within national borders (e.g., the state-of-the-art TRASE dataset do not provide any information about companies trading soybean within Brazil), which represents a limitation to more comprehensive studies targeting traceability and sustainability of national supply chains. This study highlights the importance of such markets, which should be addressed in more detail in the near future as deforestation rates are still ongoing in the Amazon and Cerrado biomes, while soybean production continues expanding^49^. Hence, we provide three specific strategies to foster transparency and sustainability in supply chains. First, initiatives such as the Transparency for Sustainable Economies (TRASE), should be designed to foster data gathering across all economic sectors and to develop information about private entities operating in Brazilian markets, a key knowledge to support policy-governance actions. Second, place-based policy prescriptions need to be implemented in specific municipalities that produce less agricultural commodities for (therefore are less committed to sustainability agreements with) international markets. Such policy prescriptions would facilitate environmental monitoring, controlling the flows of financial capital (e.g., credit), people, and agricultural commodities such as soybeans, while also developing local strategies that improve agricultural production, and reduce environmental degradation. The *Plano de Prevenção e Controle do Desmatamento na Amazônia* (PPCDAm; Plan of Prevention and Control of Deforestation in the Amazon) is an example of such place-based policies. Successfully implemented during the 2000s, it focused on municipalities that were considered hotspots of deforestation^50^. Third (and dependent on the first strategy), to avoid sourcing of agricultural products from areas under active deforestation it must be recognized that more efforts are necessary to draw attention to the trading companies purchasing soybean and other agricultural commodities not destined for international markets^24,30,31^. It is important to also note that such trade is performed by large and well-known companies that operate under less stringent sustainability standards, and are also less subjected to third-party certification bodies (including both governmental and non-governmental certification approaches). In Brazil, such certification bodies are crucial to ensure environmental policy compliance given the lack and difficulty of official surveillance in the country^51,52^. Hence, strategies similar to SM should be encouraged, where private entities that trade agricultural commodities not destined for international markets are urged to comply with environmental legislation and/or are invited to participate in sustainability agreements, while also obtain benefits such as premium prices, better access to credits, or preferential market accessibility. It is our hope that this study provides a good foundation to further untangle and better manage complex metacoupled systems for sustainable development worldwide^53^.

## Materials and methods

### Study area

The state of Mato Grosso is the largest soybean and beef producer in Brazil, with production areas in both the Amazon, Cerrado, and Pantanal biomes, and has been under huge pressure from agribusiness development, particularly over the last thirty years^32^. Our study area (Fig. 1a; covers 67 Mha representing ca. 74% of the state) includes the 128 municipalities of Mato Grosso state with soybean production. The study area excludes conservation areas (CA^54^) and indigenous territories (IT^55^) together covering 14.6 Mha (18% of the state). These areas were excluded from the analysis because they exhibit different land use/cover processes and are under different governance regimes^56^, and thus follow different land change trajectories. Our study covered the period 2004 to 2017, which represents the longest duration of soybean trade data acquired using a consistent methodology by the Transparency for Sustainable Economies (TRASE) initiative^57^. To verify these data, we accessed official public data repositories from the Brazilian Institute of Geography and Statistics (IBGE), and the Municipal Agricultural Survey (https://sidra.ibge.gov.br/pesquisa/pam/tabelas).

### Land use/land cover data

Spanning from 2004 to 2017, our dataset is composed of different data streams derived from multiple sources freely available from public data repositories (Supplementary Material, Table 6—Data sources). Deforestation was derived from the MapBiomas dataset, version 7.0. MapBiomas is a multi-institutional initiative to develop high accuracy (91.3% overall; https://mapbiomas.org/estatistica-de-acuracia) land use/cover maps of Brazil on an annual basis, from 1985 to the present, and with a 30 m pixel resolution^58^. The 16 land use/cover classes (LULC) within MapBiomas for the state of Mato Grosso were reclassified into eight classes: ‘natural forest’, ‘forest plantation’, ‘non-vegetated areas’, ‘pasture’, ‘soybean’, ‘other crops’, ‘urban area’, and ‘water’ (Supplementary Material, Table 7—Reclassified classes). The natural forest class includes the MapBiomas original classes of ‘Forest formation’, ‘Savanna formation’, ‘Wetland’, and ‘Grassland’. These classes are used by MapBiomas for deforestation analysis (https://mapbiomas.org/metodo-desmatamento), so here we adopt the same protocol. Hence, the class ‘natural forest’ was used to calculate deforestation [i.e., Natural Forest Cover Net Change (NFC2017 – NFC2004)]—the reclassified LULC classes are aggregated at municipality level, the unit of analysis in this study.

### Soybean trade variables

To achieve the main goal of our study—assessing the association between the production of soybean for different trade destinations and deforestation—we developed two metrics: proportion of soybean destined for international markets (and its complement, the proportion not destined for international markets) and soybean ‘trade destination instability’. These metrics were calculated using the TRASE Brazilian soy *v2.6* and IBGE data sources (Supplementary Material, Table 6—Data sources). We considered international trade to be the soybean produced in the state of Mato Grosso that is exported to international markets, as indicated by the TRASE dataset.

In this study we used an indirect approach to establish a binary trade destination classification (destined for international vs. domestic markets) using export data at the municipality level from TRASE (soybean exports only). This indirect approach consists in subtracting international exports from the total soybean production, on a per municipality basis. We then calculated the proportion of soybean production (during the 2004–2017 period) not destined for international markets, and its complement (i.e., production destined for international markets). In addition, to considering the importance of the temporal consistency of trade destination on environmental outcomes due to supply chains^25^, we calculated the soybean ‘trade destination instability’ of each municipality over the fifteen-year period evaluated. This metric is similar to the ‘persistence’ metric proposed by Reis et al.^25^ to analyze logistic hubs of soybean in Brazil with trade destinations. To calculate trade destination instability on a per municipality basis, we first calculated the proportion of trade not destined for international markets on an annual basis (from 2004 to 2017). We then calculated the Standard Deviation (SD) of this proportion over the fourteen-year period on a per municipality basis. This constitutes the ‘trade destination instability’, with the lower SD representing a lower instability of market destination. This ‘trade destination instability’ is a suitable measure of the inter-annual fluctuations of market destination. We set our study period to 2004–2017 since this period contains the most consistent and robust data on soybean trade, according to TRASE^57^.

While our indirect approach is different from the one adopted by TRASE to allocate production destined for international/domestic markets^57^, it is more suitable for the purposes of this study for two main reasons. On the one hand, because some soybean production originally destined for domestic markets may end up being exported to international markets through complex supply chains, allocating this proportion as completely being domestic may not be correct. On the other hand, the TRASE data representing domestic trade destination do not include all the municipalities involved in soybean production. Thus, our indirect approach not only allows obtaining a comprehensive dataset that considers all soybean production municipalities, but also separates soybean production destined, or not, for international markets. This distinction is important given that soybeans not destined for international markets could may well end up being ultimately exported to international markets through complex national supply chains that may not necessarily follow sustainability agreements or environmental certification approaches. To verify our metric of the proportion of soybean production not destined for international markets, we calculated the same variable using exclusively the information of soybean ‘domestic consumption’ available in the TRASE dataset. These values are obtained in the TRASE dataset through the use of the *supply chain mapping method*, in which the amount of soybean production at the municipality level destined for domestic consumption is calculated using a mathematical optimization process through linear programming^57^. In this process, domestic consumption at the municipality level is allocated before establishing the amount of soybean produced for a specific export trade flow, using each municipality’s soybean crushing capacity (as defined by the number of crushing and processing facilities per municipality) unless the export was identified as coming from a farm, in which case the amount of soybean assigned to this trade flow is removed from production in the municipality^57^. Comparing the values obtained through our indirect approach against those obtained only from the TRASE dataset for 2017 (which had data for 55 municipalities in Mato Grosso for domestic consumption in 2017), a 97% agreement (*Pearson* correlation—0.9701, p < 0.001) was found. Yet, our indirect approach allowed us to obtain data for the other soybean producing municipalities in Mato Grosso during the same year. Furthermore, by accounting for the TRASE data on soybean exports to international markets plus our dataset on soybean production not destined for international markets, we found a 100% agreement with the IBGE production statistics. In addition, from the total soybean production in the state of Mato Grosso, the soybean exported based on TRASE represented 92% of the soybean exported as reported by AGROSTAT (Statistics on the International Trade of the Brazilian Agribusiness) in 2017, and 98% in 2004 (i.e., the beginning of our time series). Finally, using only soybean domestic consumption from TRASE, a model developed to assess the impacts of trade destination on deforestation (see methods for this below) did not exhibit a significant relationship with deforestation (Supplementary Material, Table 5—TRASE model), while a significant relationship was found using our indirect but comprehensive trade dataset (Fig. 2). Although developed using an indirect approach, our trade destination dataset shows considerable consistency with (*i*) the TRASE domestic consumption, and with (*ii*) the official statistics at the state level, while also allows analyzing the effects of trade destination on deforestation throughout all soybean producing municipalities in the state of Mato Grosso.

### Spatial modelling and statistics

We evaluated the association between deforestation (our dependent variable) and the ‘proportion of soybean production not destined for international markets’ (our independent variable) on a per municipality basis, using a spatial autoregressive model with a spatially lagged dependent variable (SARlag), along with descriptive and inferential statistics. The model tested our independent variable together with a set of additional factors: change in total ‘soybean production’, ‘slope’, pastureland area in 2004 ('pastureland’), number of formal jobs in agriculture (‘Agri-job’), number of animals per hectare (‘cattle density’), and the ‘soybean trade instability’. The soybean production [14-year delta (i.e., Δ*t14* = 2004–2017)] was applied as an additional factor, since one could hypothesize that changes in the amount of soybean produced, which is driven by national and international demands^37,59^, exert an influence on deforestation, regardless of market destination. Such demands are generated by economic development, dietary changes toward more meat, population increases, and even faster increases in the number of households due to factors such as divorce^60–62^. In addition, raising cattle for beef production is known as an important force of deforestation in tropical regions^43^ and here we used the mean density of cattle (number of animals/municipality area) between 2004 and 2017 and pastureland area in 2004 as control variables. Number of jobs in the agricultural sector is a reliable proxy to measure the socioeconomic standing of a given production region as relating to land change dynamics^59,63,64^, and for agglomeration economies^65^. Here we use the mean values for the period. Topography plays an important role in the agricultural expansion of Brazil, especially in large-scale agricultural commodity production areas, such as in Mato Grosso^66^. Hence, our model used slope (in degrees) to represent a key biophysical controlling factor. Slope data were derived from the TOPODATA geomorphometric information system of Brazil^67^.

An ordinary least square (OLS) regression was first applied, followed by a Lagrange Multiplier (LM) test for the diagnosis of spatial dependence^68^. Accounting for the spatial trends and dependence, in the SARlag model the spatial autocorrelation term is associated with the dependent (i.e., response) variable^59^. This approach is suitable for modelling forest dynamic processes as previous studies have pointed out that in agricultural frontiers, deforestation and crop expansion are influenced by diffusion of information, land prices, and logistic development^18,37^. This suggests a process of contagion (i.e., path dependence) of spread from one cultivated area to the edges of nearby natural forests^37,59^. Our SARlag model and LM tests used a first-order spatial weighed matrix with the neighbors defined by the Queen contiguity approach^69,70^. The variance inflation factor (VIF) test was applied (over the OLS regressions) to avoid multicollinearity among explanatory variables. A VIF value of 5 was set as the limit to accept an independent variable, following previous studies^71^. Finally, as described above, we run an additional model with the variables ‘proportion of soybean production not destined for international markets’ and ‘soybean trade instability’ derived using only the TRASE dataset (i.e., ‘domestic consumption’ data derived from TRASE).

To explore the relationships among the soybean trade metrics with each other and with other variables (e.g., soybean farm size), we performed correlation tests using Spearman rank (after Shapiro–Wilk test) in the case of non-normally distributed data, or using Pearson in the case of normality^43^. Soybean farm size has previously been shown to be a key driver in shaping Brazilian agricultural systems^26,33^, with possible effects on land use decisions, thus ultimately affecting landscape outcomes. Here we estimated farm size as the mean area of soybean cultivated land per producer in each municipality, following the approach developed by Silva et al.^33^. Mean farm size was calculated for the years 2006 and 2017—the years of the last two agricultural censuses of the IBGE dataset (Supplementary Material, Table 6—Data sources). Considering the well-known land process of indirect land use change with pasture primarily replacing natural forest areas and being followed by soybean area expansion^24,41^, we explored the spatial association between the ‘proportion of soybean production not destined for international markets’ and ‘trade destination instability’ with pastureland expansion and cattle statistics [‘cattle density’, and cattle herd expansion over the study period (Supplementary Material, Table 6—Data sources)].

## Supplementary Information

Supplementary Information.

## Data Availability

The datasets generated and/or analyzed during the current study are freely available through their respective public repositories [Repository names and links for access are provide in Supplementary Table 6—Data Sources].
