# Supplementary material for: Complex relationships between soybean trade destination and tropical deforestation
Source: Sci Rep. 2023 Jul 12;13:11254. doi: 10.1038/s41598-023-38405-1 (PMC10338464; doi:10.1038/s41598-023-38405-1)
Supplement: Supplementary file 1 — Supplementary Information. [file 41598_2023_38405_MOESM1_ESM.pdf]

Supplementary Material

Complex Relationships Between Soybean Trade Destination and Tropical Deforestation

1 Supplementary Tables

Supplementary Table 1. Change Matrix for 2004-2011—Mato Grosso. Units are hectares.

	Natural forest	Pasture	Soybean	Other crops	Forest plantation	Urban area	Non-vegetated areas	Water	**TOTAL 2004**
Natural forest	37095571	2743853	281553	332642	6502	2328	15790	111775	**40590018**
Pasture	1161319	17031314	843678	1029837	35898	5248	11197	3515	**20122008**
Soybean	12804	81087	4273301	603746	5151	958	1542	372	**4978965**
Other crops	113398	482517	1115427	1374340	5994	1241	4879	657	**3098458**
Forest plantation	938	679	741	343	23157	0.26	1	7	**25869**
Urban area	38	44	12	41	0.35	84110	18	8	**84275**
Non-vegetated areas	11144	15737	11186	15433	181	1318	10043364	829	**156264**
Water	82904	1933	22	449	4	21	1971	437673	**524980**
**TOTAL 2011**	**384781193**	**20357169**	**6525923**	**3356833**	**76890**	**95229**	**135835**	**554839**	

Supplementary Table 2. Change Matrix for 2011-2017—Mato Grosso. Units are hectares.

	Natural forest	Pasture	Soybean	Other crops	Forest plantation	Urban area	Non-vegetated areas	Water	**TOTAL 2011**
Natural forest	36065026	1845953	211921	251587	15375	1852	19589	66826	**38478129**
Pasture	1165301	16299299	1599995	1236821	31728	5313	14643	4066	**20357166**
Soybean	7972	47738	6206520	256436	5011	1302	860	90	**6525929**
Other crops	105511	539367	1464713	1202979	35224	1893	6491	659	**3356835**
Forest plantation	1860	1098	1359	1694	70848	2	29	0	**76890**
Urban area	41	58	23	39	0	95030	14	23	**95229**
Non-vegetated areas	6418	10441	6993	8920	592	744	100378	1350	**135835**
Water	115221	1373	18	546	12	23	1276	436369	**554839**
**TOTAL 2017**	**37467351**	**18745326**	**9491541**	**2959023**	**158790**	**106159**	**143279**	**509383**	

Notes on Supplementary Tables 1 and 2

Supplementary tables are matrices of change in land use/cover (LULC) between two points in time in hectares, with column and row totals. Areas of individual LULC classes for the initial point in time (e.g. in Supp. Table 1 this is 2004) are located in the vertical axis of the Table. Hence, the LULC area for each class for the initial time point is shown in the final column of the table. Correspondingly, areas of individual LULC classes for the second point in time are located in the horizontal axis, with the final row showing the total area for each class in the late point in time. The values in each cell in the Table indicate the amount of land moving from one class to another. For example, Supplementary. Table 1 shows 281,553 ha changed from Natural Forest in 2004 to Soybean in 2011 and 1,599,995 ha from Pasture to Soybean.

Supplementary Table 3. Spatial regression model results.

	**Model coefficients**
**Independent variables**	**Deforestation 2004—2017**
Constant	9436.54
Proportion not destined for international markets	172.762*
Soybean trade instability	57.0596
Soybean production ($\Delta t14$)	0.0298*
Pastureland 2004	0.0554***
Agricultural job	-0.953
Cattle density	-23186.7***
Slope	-985.3*
W (lag model)	0.3247**
R-squared	0.4495

* indicates significance at the 95% confidence level, ** =99%, and *** = 99.9%. n = 128. *W* is the spatial lag term of the dependent variable (its coefficient parameter, rho, reflects the spatial dependence inherent in the data).

Supplementary Table 4. Correlations.

Correlation test	Coefficient	p-value
Proportion not destined for international markets *vs* soybean trade instability	-0.4998	p < 0.001 (Spearman)
Proportion not destined for international markets *vs* cattle herd change (2004—2017)	0.2529	p < 0.05 (Pearson)
Proportion not destined for international markets *vs* mean cattle density (2004—2017)	0.2535	p < 0.01 (Spearman)
Proportion not destined for international markets *vs* pastureland area change (2004—2017)	0.2625	p < 0.01 (Spearman)
Proportion not destined for international markets *vs* mean farm size	-0.4066	p < 0.001 (Spearman)
soybean trade instability *vs* mean farm size	0.2106	p < 0.05 (Spearman)

Supplementary Table 5. TRASE model—Spatial regression model results.

	Model coefficients
**Independent variables**	**Deforestation 2004—2017**
Constant	26373.3**
Proportion not destined for international markets	22.2061
Soybean trade instability	17.5562
Soybean production ($\Delta t14$)	0.0294**
Pastureland 2004	0.0529***
Agricultural job	-2.9583
Cattle density	-24167.1***
Slope	-1211.14*
W (lag model)	0.1712
R-squared	0.4313

* indicates significance at the 95% confidence level, ** =99%, and *** = 99.9%. n = 112. *W* is the spatial lag term of the dependent variable (its coefficient parameter, rho, reflects the spatial dependence inherent in the data).

Supplementary Table 6. Data Sources.

Datasets	Geographical scale	Source
Crop production	Municipality	IBGE/PAM (table 1612) - https://sidra.ibge.gov.br/tabela/1612
Soybean trade/exports	Municipality	TRASE/Soy - https://trase.earth/explore
LULC	Municipality	MapBiomas - https://mapbiomas.org/download
Cattle herd	Municipality	IBGE/PPM (table 3939) - https://sidra.ibge.gov.br/tabela/3939
Gross domestic product	Municipality	IBGE/PIBM (table 5938) - https://sidra.ibge.gov.br/tabela/5938
Formal jobs	Municipality	IBGE/CNAE 2.0 (table 6449) - https://sidra.ibge.gov.br/Tabela/6449
Soybean farm size	Municipality	Silva et al. 2020 - https://doi.org/10.3390/land9110422
Slope (topography)	Brazil (pixel level)	Valeriano 2008 - http://www.dsr.inpe.br/topodata/documentos.php
Transportation Cost	Brazil (pixel level)	Victoria et al. 2021 - https://doi.org/10.1016/j.dib.2021.107070

Supplementary Table 7. Reclassified Classes

MapBiomas *v7.0*		Reclassification	
**Class**	**ID**	**New class**	**ID**
Forest Formation	3	Natural Forest	1
Savanna Formation	4		
Wetland	11		
Grassland	12		
Pasture	15	Pasture	2
Soybean	39	Soybean	3
Sugar cane	20	Other Crops	4
Mosaic of Uses	21		
Cotton	62		
Other Temporary Crops	41		
Forest Plantation	9	Forest Plantation	5
Urban Area	24	Urban Area	6
Mining	30	Non-Vegetated Areas	7
Other non Vegetated Areas	25		
Rocky Outcrop	29		
River, Lake and Ocean	33	Water	8

2 Supplementary Figures

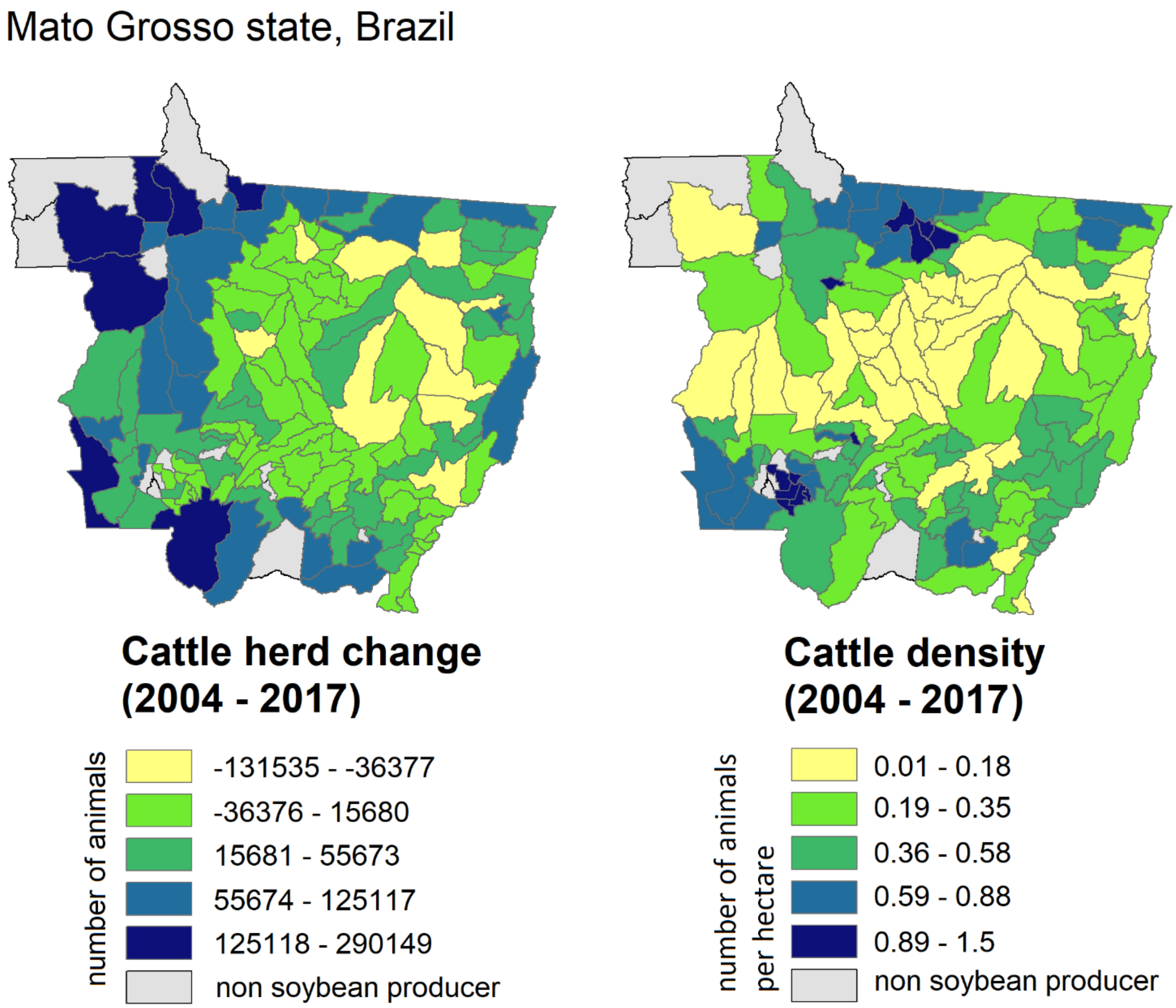

Supplementary Figure 1. Geographic distribution of cattle statistics (change in the cattle herd between 2004 and 2018, and mean cattle density for the 2004—2018 period) per each soybean producing municipality of Mato Grosso state, Brazil.

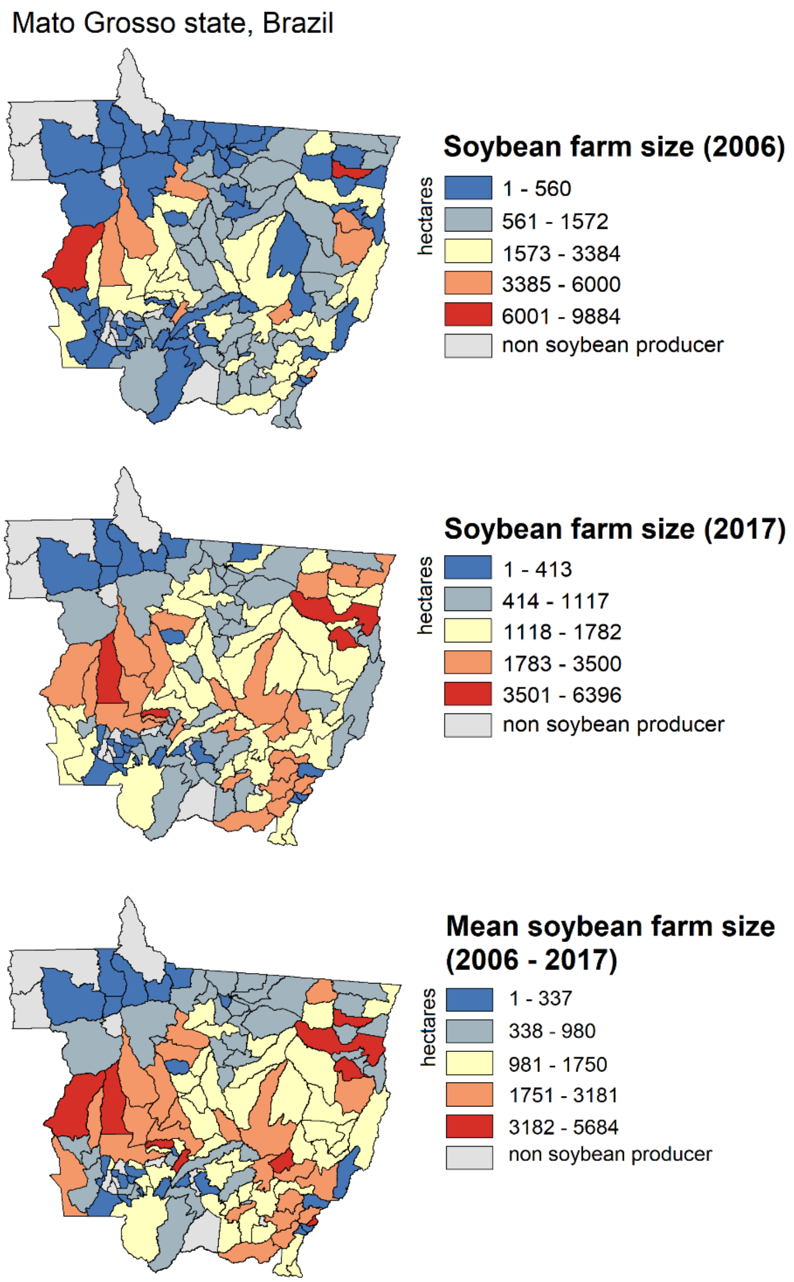

Supplementary Figure 2. Soybean farm size in municipalities of Mato Grosso state, Brazil. Mean farm size was calculated as the average of the soybean farm size for the years of 2006 and 2017 (data from Silva et al., 2020 and derived from the IBGE agricultural Censuses of 2006 and 2017).
